# Plugging the Attention Deficit: Perceptual Load Counters Increased Distraction in ADHD

**DOI:** 10.1037/neu0000020

**Published:** 2013-11-11

**Authors:** Sophie Forster, David J. Robertson, Alistair Jennings, Philip Asherson, Nilli Lavie

**Affiliations:** 1 Department of Clinical, Educational, and Health Psychology, University College London, London, UK; 2 Institute of Cognitive Neuroscience and Research Department of Cognitive, Perceptual, and Brain Sciences, University College London, London, UK; 3 MRC Social, Genetic, and Developmental Psychiatry Centre, Institute of Psychiatry, King’s College London, London, UK

**Keywords:** attention-deficit hyperactivity disorder, ADHD, distraction, perceptual load, inattention

## Abstract

***Objective:*** Increased vulnerability to extraneous distraction is a key symptom of Attention-Deficit Hyperactivity Disorder (ADHD), which may have particularly disruptive consequences. Here we apply Load Theory of attention to increase understanding of this symptom, and to explore a potential method for ameliorating it. Previous research in nonclinical populations has highlighted increased perceptual load as a means of improving the ability to focus attention and avoid distraction. The present study examines whether adults with ADHD can also benefit from conditions of high perceptual load to improve their focused attention abilities. ***Method:*** We tested adults with ADHD and age- and IQ-matched controls on a novel measure of irrelevant distraction under load, designed to parallel the form of distraction that is symptomatic of ADHD. During a letter search task, in which perceptual load was varied through search set size, participants were required to ignore salient yet entirely irrelevant distractors (colorful images of cartoon characters) presented infrequently (10% of trials). ***Results:*** The presence of these distractors produced a significantly greater interference effect on the search RTs for the adults with ADHD compared with controls, *p* = .005, η_p_^2^ = .231. Perceptual load, however, significantly reduced distractor interference for the ADHD group and was as effective in reducing the elevated distractor interference in ADHD as it was for controls. ***Conclusions:*** These findings clarify the nature of the attention deficit underlying increased distraction in ADHD, and demonstrate a tangible method for overcoming it.

Symptoms of inattention and distractibility are key diagnostic criteria for Attention Deficit Hyperactivity Disorder (ADHD) and appear to form the most pervasive component of the disorder; inattentive symptoms persist into adulthood more commonly than symptoms of hyperactivity-impulsivity (Wilens, Faraone & Biederman, 2004). Such symptoms can be highly disruptive in many daily life contexts from the workplace to the drive home from work. Indeed, many disadvantages reported by adults with ADHD, such as academic failure, workplace problems, and increased risk of car accidents (Faraone et al., 2000), have also been associated with increased daily life distractibility in nonclinical populations (Forster & Lavie, 2008b, for review), suggesting that symptoms of distraction may (at least partially) mediate the risk of functional impairments in ADHD.

The present study applies the Load Theory of Attention and Cognitive Control (Lavie, 1995; Lavie et al., 2004) to enhance understanding of the increased daily life distraction in ADHD, and to examine ways to ameliorate this. According to Load Theory, a key determinant of the ability to focus attention while avoiding distraction is whether the task being performed involves a sufficiently high “perceptual load” to fill perceptual capacity. Perceptual load has been operationally defined as either the quantity of stimuli requiring perceptual processing, or the complexity of perceptual judgments (Lavie, 2005). Tasks involving low load (e.g., involving few items, and simple judgments) are argued to leave spare capacity which spills over, resulting in involuntary processing of distractors. In this respect low load tasks confer vulnerability to distraction and necessitate reliance on executive “late selection” mechanisms to minimize interference. On the other hand, when the task processing involves high load (e.g., requiring complex perceptual judgments or searching among many items), using up the available perceptual capacity, perception of distractors is reduced or even eliminated. Thus, higher levels of perceptual load engender more efficient “early selection” of the task stimuli and reduce vulnerability to distraction.

In support of Load Theory are abundant demonstrations that increased perceptual load in the task results in reduced distractor processing throughout visual cortex, leading to reduced distractor interference across a range of distractor types (Lavie, 2005, for review). Thus, increasing perceptual load appears to be a powerful method for reducing distraction in nonclinical populations. However, it remains to be established whether increasing perceptual load would be equally effective in reducing the increased level of distraction experienced in ADHD.

The neural mechanisms underlying ADHD are heterogeneous and remain poorly characterized, although deficits in frontal-executive processes are proposed as one of the major sources of symptoms and impairments seen in ADHD (e.g., Bush, Valera & Seidmanm, 2005). Underrecruitment of the frontal-executive mechanisms, thought to underlie late selection (Lavie et al., 2004), may therefore play a key role in the disruption underlying distraction in ADHD. It remains unknown, however, whether deficits in ADHD may also involve early selection mechanisms or whether these remain intact.

Two previous studies (Huang-Pollock, Nigg, & Carr, 2005; Chan et al., 2009) examining perceptual load effects within response-competition tasks provide encouraging preliminary support for the hypothesis that early selection is intact in ADHD. Both studies found that perceptual load reduced distractor interference in children with ADHD and controls alike. However, neither study reported increased overall distractor interference in the ADHD group, even under low perceptual load (when risk of distraction is greatest). Huang-Pollock et al. (2005) found no group differences, whereas Chan et al. (2009) found a laterality bias, whereby children with ADHD experienced either more or less interference than controls depending on whether distractors were presented in the right or left visual field. Thus, as perceptual load effects have not yet been tested within a paradigm sensitive to reveal heightened distraction in ADHD, it is unclear whether elevated distraction in ADHD can be efficiently reduced by a potentially intact early selection mechanism.

To this end, the present study sought first to identify a laboratory measure sufficiently sensitive to reveal increased distraction in ADHD. Although the response-competition task is an established and widely used measure of distractor interference, we argue that this task may not be the most appropriate index for the symptom of irrelevant distraction in ADHD. Within this task, distraction is indexed by the performance decrement in the presence of a distractor associated with a competing response to the target. This is assessed against the baseline of performance in the presence of a response-compatible or response-neutral distractor. In this respect distraction in this paradigm does not reflect the extent of noticing and paying attention to an irrelevant distractor (in other words the extent of inattention), but instead the extent to which response associations of different distractor stimuli affect the target response. The defining role of distractor–response associations in this measure presents two limitations in indexing irrelevant distraction in ADHD. First, in the light of proposals that disruption to inhibitory mechanisms is one of the deficits underlying ADHD (e.g., Halperin & Schulz, 2006; Johnson, Wiersema, & Kuntsi, 2009), it is unclear whether any findings of inflated response-competition effects in ADHD can be attributed to failures of attention, as opposed to failures of response inhibition. In addition, the fact that response-competition effects indicate slowing of target responses attributable to competing response-incompatible (vs. response-compatible or response-neutral) distractors confines the scope of this measure to a very particular class of distractors, namely those that have specific associations with the task responses. This does not seem to capture the clinical symptoms of distraction by “extraneous stimuli” (as per DSM criterion), which are by definition task-unrelated and hence not particularly associated with one task response or the other.

Indeed, despite its prevalent use, previous studies of ADHD using variants of the response-competition task have produced somewhat inconsistent results; although some studies find evidence of increased interference in the ADHD group (e.g., Crone, Jennings, & van der Molen, 2003; Jonkman et al., 1999; Konrad et al., 2006; Vaidya et al., 2005), others did not (e.g., Booth et al., 2007; Brodeur & Pond, 2001; Chan et al., 2009; Chang, Davie, & Gavin, 2009; Geurts et al., 2008; Herrmann et al., 2010; Huang-Pollock et al., 2005; McLoughlin et al., 2009). Studies in natural settings, however, support the clinical picture that sources of distraction in ADHD tend to have little relevance to any current task. For example, while following a route through a zoo, children with ADHD were more likely than controls to veer off the designated route toward animals in other routes (Lawrence et al., 2002). Another study found children with ADHD to be significantly more distracted from watching TV by the presence of irrelevant yet appealing toys (Landau, Lorch, & Milich, 1992). Note also that in both of these examples, as is often the case in daily life, the stimuli that people fail to ignore despite their irrelevance to the task are highly salient (both “eye-catching,” and meaningful). In contrast, response-competition tasks typically use rather less salient distractor stimuli, such as letters, or simple shapes. Thus we anticipated that a more general measure of interference from salient yet entirely task-irrelevant distractors might prove more sensitive to reveal the distraction deficit in ADHD.

Finally, we note that the majority of previous investigations into attention in ADHD have studied child populations. As both early and late attentional selection processes are subject to significant developmental changes (Huang-Pollock, Carr & Nigg, 2002), it is important to establish whether any disruption to these processes reflects a core deficit (consistent with the persistence of inattentive symptoms into adulthood, Wilens, Biederman, & Spencer, 2002) as opposed to simply a developmental delay.

We therefore compared the performance of adults with ADHD with that of age-matched control participants on a recently established measure of irrelevant distraction (Forster & Lavie, 2008a, 2008b), designed to reflect a more general form of distraction than that measured in the response-competition task. Participants were asked to identify which of two possible “target” letters was present in a central letter search display, while ignoring any images presented outside the display. A colorful distractor image depicting a famous cartoon character (e.g., Spiderman) was presented in the periphery on the minority (10%) of trials. Note that, like the animals and toys that distracted ADHD groups in the naturalistic studies described above, the distractor in our measure was entirely irrelevant to the letter-search task: being visually dissimilar to the search-task stimuli, presented in an irrelevant location, and bearing no relation to the task through content and meaning, or task responses. Distractor interference was indexed simply by the increase in reaction times (RTs) for the presence versus absence of the images (following Forster & Lavie, 2008a, 2008b).

To test our claim that the elevated distraction in ADHD can be ameliorated under conditions of high perceptual load, we manipulated perceptual load by changing the similarity between the target and the other search letters. In the low load condition the other letters were five small ‘o’s, whereas in the high load condition they were five angular letters (see Figure 1). This manipulation changed the complexity of perceptual judgments as per one of the main operational definitions of perceptual load described earlier. We predicted that, in accordance with the diagnostic criteria of increased distraction, compared with age-matched controls the ADHD group will show increased RT interference from the task-irrelevant distractors. Importantly, we examined whether perceptual load would be as effective in reducing this heightened distraction for adults with ADHD, as in reducing distraction for the control group.[Fig-anchor fig1]

## Method

### Participants

Thirty-four age-matched adults participated in the study: 17 adults with ADHD and 17 volunteer controls. Participants in the ADHD group were recruited via the Maudsley Hospital Adult ADHD Clinic and local ADHD support groups. All ADHD participants were diagnosed in the U.K. by a psychiatrist who prescribed stimulant medication treatment (or in the case of one participant, had offered to prescribe stimulant treatment but this was declined). The U.K. diagnostic standards are conservative, with the diagnosis requiring evidence of ADHD in childhood and persistence of sufficient symptoms and impairment into adulthood. The standard diagnostic procedure uses a clinical diagnostic interview and detailed developmental account, with informants included if they are available. Fifteen of the ADHD participants were taking prescribed stimulant medication (methylphenidate, atomoxetine, amphetamine and dextroamphetamine)—these participants were tested after a 24-hour medication washout period.

On the day of testing, participants completed the Barkley and Murphy (2006) Current Symptoms Rating Scale—this is a Likert-type rating scale in which each *Diagnostic and Statistical Manual of Mental Disorders*, fourth edition (*DSM–IV*) ADHD that symptom occurs is rated as occurring from *never or rarely* (0) to *very often* (3). As per the *DSM–IV* criteria, half of the items relate to inattentive symptoms, and half relate to hyperactive-impulsive symptoms. Scores can be computed using two methods (Barkley & Murphy, 2006): First, the number of symptoms reported as occurring “often” or “very often” may be counted. Using this method, all participants in the ADHD group met the threshold of six or more current inattentive symptoms at the time of testing. Eleven of these participants additionally met the threshold of six of more hyperactive-impulsive current symptoms. To obtain a continuous measure of the degree of current symptoms, responses to each item can be also summed (Summary Scores). Mean Symptom Counts and Summary Scores are presented in Table 1.[Table-anchor tbl1]

Control participants were recruited via the University College London volunteer participant pool. Participants were recruited by age, to match the ADHD group. The ADHD and control groups showed similar IQ scores (*t* < 1 for group differences, see Table 1; IQ scores were unavailable for four of the ADHD group—excluding these participants from analysis produced an identical pattern of results), assessed using the Matrix Reasoning and Vocabulary subtests of the Wechsler Abbreviated Scale for Intelligence (WASI; Wechsler, 1999). Participants in both groups had normal range IQ and normal or corrected to normal vision. Additional exclusion criteria were any diagnosed learning disability, history of neurological disorder, severe head injury or diagnosed neuropsychological impairment, current diagnosed axis I or axis II *DSM–IV* psychiatric disorder (other than ADHD for the ADHD group), or current use of psychoactive medication (other than prescribed ADHD medication for the ADHD group). All control participants reported no history of ADHD and reported fewer than the threshold of six symptoms on both the hyperactive-impulsive and inattentive scales of Barkley and Murphy’s Current Symptoms Rating Scale. The data of one control participant were omitted from analysis for having a percentage error rate over three standard deviations greater than the mean among control participants in the high load condition.

### Stimuli and Procedure

**Figure 1 fig1:**
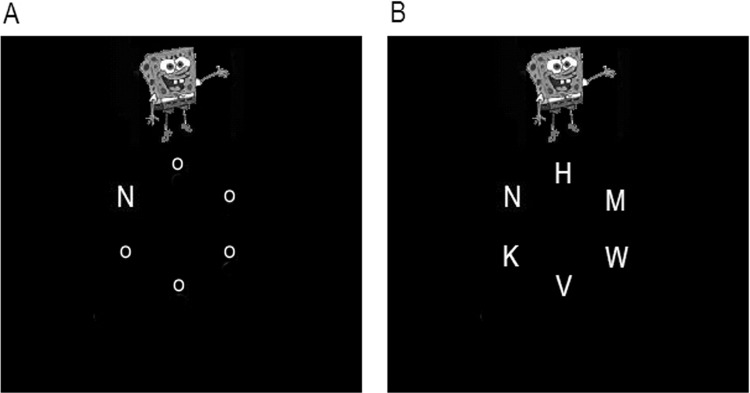
Example “distractor present” letter search stimulus displays in (A) low perceptual load and (B) high perceptual load. All stimuli were presented on a black background, with all letter stimuli presented in light gray. The letter circle radius subtended 1.6° degrees of visual angle, with the target letters subtending 0.6° by 0.4°. In the low load condition (A), nontarget positions were occupied by small ‘o’s (0.15° by 0.12°). In the high load condition (B) the five nontarget positions were occupied by heterogeneous angular letters of the same dimensions as the target-randomly chosen from the set K, V, W, Z, M, and H. On distractor-present trials (10% trials), a full-color cartoon image (subtending 2.8–4° vertically by 2.8–3.2° horizontally) was presented 4.6° from fixation with a minimum of 0.6° edge to edge from nearest letter stimulus. Each distractor image was drawn with equal probability from the following set of cartoon characters: Superman, Spiderman, Spongebob Squarepants, Pikachu, Mickey Mouse, and Donald Duck.

Participants were tested individually in a quiet room, at viewing distance of approximately 57 cm (maintained using a chinrest) from a 15 in. monitor. Each trial began with a 500-ms presentation of a fixation cross, immediately followed by a 200-ms presentation of six letters arranged to form a circle (see Figure 1 for example stimulus displays). The task was to identify which of two possible target letters (X or N) was present, pressing the 0 key for ‘X’ and the 2 key for ‘N.’ Participants were emphatically instructed to respond as fast as possible *while still being accurate*, ignoring any stimuli except for the letter-search set.

On “no distractor” trials (90% of trials) the letter circle display appeared alone. On 10% of trials a distractor cartoon image was presented either above or below the letter circle display, remaining onscreen until the end of the trial. Each trial ended either upon response, or (if no response was made) after 2000 ms. A tone sounded for incorrect or missed responses. Participants completed two blocks of 12 practice trials, followed by eight blocks of 60 trials. Load was manipulated between blocks in the order Low-High-Low-High-Low-High-Low-High or High-Low-High-Low-High-Low-High-Low (counterbalanced between participants). The first three trials of each block were treated as warm up trials and never contained a distractor.

## Results

Mean RTs and percentage error rates were calculated as a function of group and experimental condition (see Table 2).[Table-anchor tbl2]

### RTs

**Figure 2 fig2:**
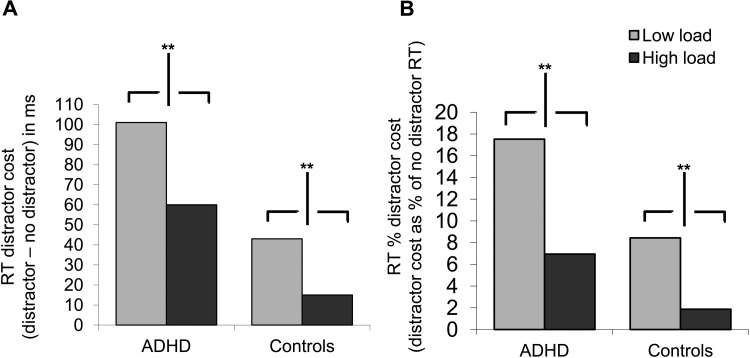
Mean RT distractor costs as a function of load and group. ** *p* < .01.

RTs of less than 100 ms or greater than 1500 ms (<6% responses for the ADHD group, < 2% responses for controls), and incorrect responses, were excluded from RT analysis. Mixed model ANOVA (group × load × distractor condition) revealed a main effect of load, *F*(1, 31) = 208.64, *MSE* = 9665.84, *p* < .001, η_p_^2^ = .871, 95% CI [212.91–281.57] (see Figure 2), confirming that our manipulation of load was effective in increasing processing demands. There was no interaction between load and group, *F*<1, η_p_^2^ = .001, thus the increase in processing demands was similar for ADHD and control groups. There was also a main effect of distractor presence, *F*(1, 31) = 42.22, *MSE* = 2355.03, *p* < .001, η_p_^2^ = .577, 95% CI [36.38–75.01], reflecting significantly slowed RTs in the presence (vs. absence) of the distractor. Critically, there was a significant interaction between group and distractor condition, *F*(1, 31) = 9.29, *p* = .005, η_p_^2^ = .231. As can be seen in Figure 2, the ADHD group showed significantly greater RT interference from the distractors, compared with controls. This indicates that our task is sensitive to detect inflated distraction in ADHD.[Fig-anchor fig2]

Distractor presence also significantly interacted with load, *F*(1, 31) = 7.94, *MSE* = 1206.39, *p* = .008, η_p_^2^ = .204, reflecting that distractor interference was reduced under high load, *t*(32) = 4.04, SEM = 9.55, *p* < .001, *d* = 1.43, 95% CI [19.09–58.00], compared with low load, *t*(32) = 6.01, SEM = 12.65, *p* < .001, *d* = 2.12, 95% CI [47.07–98.62]. Importantly, there was no three-way load × distractor × group interaction, indicating that load was equally effective in decreasing distractor interference for all participants, *F* < 1, η_p_^2^ = .010.

In keeping with previous findings of slower RTs in ADHD (e.g., Klein et al., 2006) the ADHD group showed slower RTs overall, *F*(1, 31) = 6.751, *MSE* = 40635.62, *p* = .014, η_p_^2^ = .179. However, differences in overall RT did not account for the increased distractor costs. An additional ANOVA on the percentage increase in RT for distractor trials compared with no distractor trials, in high and low load, confirmed significantly greater percentage distractor costs in the ADHD group, *F*(1, 31) = 7.85, *MSE* = 105.55, *p* = .009, η_p_^2^ = .202, 95% CI [1.60–11.46]. It is striking to note that even when baseline RT was controlled for, the distractor costs of the ADHD group were more than twice those seen for control group. Most importantly, however, despite their greatly increased levels of distraction, even when overall RT slowing is accounted for it is clear that the ADHD group were able to benefit from a significant reduction in distraction under high load, *t*(16) = 3.13, SEM = 3.38, *p* = .006, *d* = 1.57, 95% CI [3.412–17.75]. As can be seen in Figure 2, load was just as effective in reducing distraction in adults with ADHD as for controls (for interaction of load by group *F*(1, 31) = 1.12, *p* > .29, η_p_^2^ = .035). In fact increasing load compensated for the increased distractor costs of the ADHD participants, to the extent that the high load distractor costs of the ADHD group were of a similar level to those experienced by the control group under low load (*t* < 1 for the difference, *d* = 0.20).

**Figure 3 fig3:**
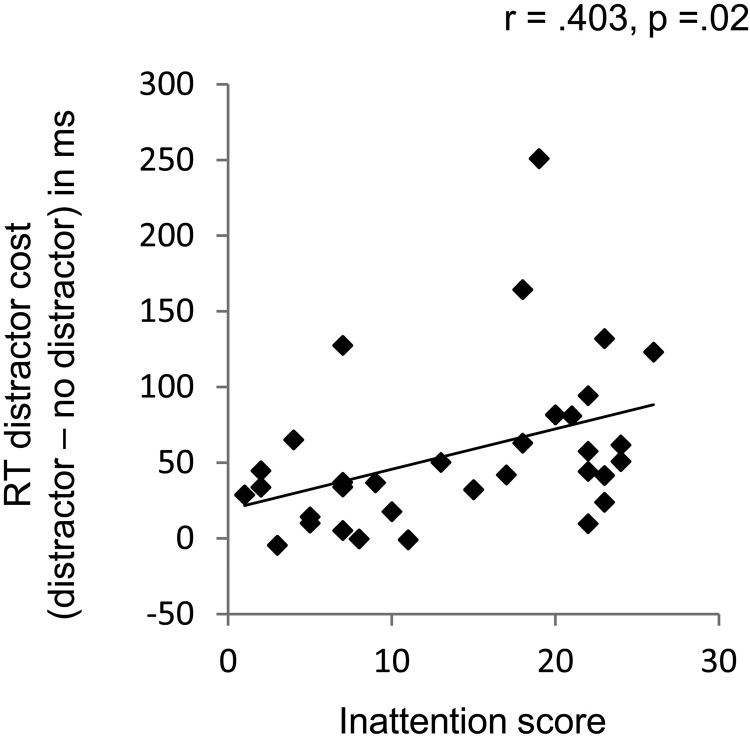
Individual differences in RT cost as a function of inattention scores.

Finally, we examined the extent to which specific symptoms of ADHD predicted distractor interference. A multiple regression, conducted on inattentive and hyperactive-impulsive Summary Scores for both ADHD and control participants, revealed that inattentive scores alone significantly predicted overall distractor cost (see Figure 3): For the model *r*^2^ = .203, *F*(1, 31) = 3.83, *p* = .033 (inattentive *b* = .709, *p* = .023; hyperactive-impulsive *b* = −.367, *p* = .225).[Fig-anchor fig3]

### Percentage Error Rates

Although our use of a RT task, with an emphasis on performance speed, meant that RT was our main measure, the error rates also showed a main effect of distractor presence, *F*(1, 3) = 4.28, *MSE* = 54.03, *p* = .047, η_p_^2^ = .121, 95% CI [.09–5.28], reflecting more errors in the presence of a distractor. A main effect of load on error rates was also found, *F*(1, 3) = 58.87, *MSE* = 105.61, *p* < .001, η_p_^2^ = .655, 95% CI [9.95–17.78]. There was no interaction between load and distractor presence, although the trend mirrored the pattern found on RTs, *F*(1, 31) = 1.12, *MSE* = 29.87, *p* = .30, η_p_^2^ = .023.

A significant main effect of group was found on errors, *F*(1, 31) = 6.31, *MSE* = 205.10, *p* = .017, η_p_^2^ = .169, 95% CI [1.18–11.35], qualified by a load x group interaction, *F*(1, 31) = 5.93, *p* = .021, η_p_^2^ = .160, reflecting more errors among ADHD participants compared with controls, especially under high load. There was no interaction between group and distractor presence, or three-way load × distractor presence × group interaction, *F*s < 1, η_p_^2^ = .023 and η_p_^2^ = .022, respectively.

## Discussion

The present study establishes two key findings: i) Adults with ADHD show more than double the distractor interference compared with controls; the increase in RT in the presence of distractors is more than twice the increase seen in the control group. Furthermore, the difference in distractor costs was retained after controlling for the overall slower RTs seen in the ADHD group. This establishes our task as a sensitive measure of a distraction deficit in adults with ADHD. The magnitude of distractor costs was specifically related to the degree of inattentive symptoms reported, suggesting that our task may provide a valid measure of the ADHD inattentive symptoms related to distractibility. ii) The greater levels of distraction in ADHD can be reduced with higher perceptual load in the task. Indeed, perceptual load was equally effective in reducing distraction for adults with ADHD as it was for controls. This finding points to an intact early selective attention mechanism and suggests that distraction in ADHD results from disruption to later attention mechanisms, such as the efficiency of executive cortical control. As mentioned in the Introduction, Load Theory proposes that high perceptual load can reduce distractor interference simply by filtering distractor stimuli from perception. Efficient cortical executive control is only needed then when this early attentional selection fails. It therefore follows that facilitating early selection with high perceptual load can compensate for late selection/executive control deficits that otherwise lead to increased distraction in ADHD.

The load effects we identified may also account for the “hyper-focusing” presentation that has been observed in the clinical picture of ADHD (e.g., see Schecklmann et al., 2008; Asherson, 2005). Patients with ADHD often describe periods of intensive concentration on certain interesting tasks (e.g., video games, Internet searching, fast competitive sports) associated with diminished perception of the environment. We note that in addition to potentially increased interest, the aforementioned tasks also typically involve high level of perceptual load (i.e., the rapid presentation of multiple moving stimuli). Our findings indicate that under such conditions people with ADHD may be fully engaged in task processing, with reduced perception of other stimuli. Thus, although these two clinical manifestations of distractibility on some tasks and hyper-focusing on other tasks may appear seemingly paradoxical, our study clarifies one factor that may determine which manifestation will occur—namely, perceptual load. Of course, this does not preclude the potential role of other factors such as the rewarding nature of the stimulus and related motivational factors. However, we speculate that the phenomenon of hyper-focus in ADHD might relate specifically to tasks with a high level of load, where distraction is reduced.

Our findings also have potential implications for developing interventions to manage the highly disruptive symptom of distraction. As mentioned above, increased daily life distraction has been directly linked to a number of the negative outcomes, such as failure at school or work, or being accident prone, which are also strongly associated with the inattention symptoms (including distractibility) of ADHD. Our results suggest that by increasing perceptual load, for example in educational material (e.g., presenting periodic tables with a higher visual load), the levels of distraction in individuals with ADHD could be reduced to nonclinical levels; although note that non-ADHD individuals would also be expected to benefit to an equivalent degree from the use of higher visual load materials.

With respect to our first key findings of substantially higher distractor interference effects in adults with ADHD, compared with matched controls, which are correlated with ratings of inattentive symptoms, these suggest our RT interference measure can serve as a distractor assessment tool that provides an objective proxy for less tangible symptoms of inattention, such as failing to give close attention to work, being easily distracted, and engaging in mind-wandering (Forster & Lavie, under revision). Several aspects of our task may have increased its applicability to clinical forms of distraction reported in ADHD. Unlike previous paradigms (which have failed to produce consistent evidence of elevated distraction), our paradigm is designed to measure distraction from the presence of distractors that are entirely irrelevant to the task and can therefore generalize across different tasks and contexts (see Forster & Lavie, 2008b, 2011). Indeed it has been found that although children with ADHD do not show greater interference (vs. controls) from response-incongruent versus neutral pictorial distractors, they do show increased interference from the presence versus absence of any distractor (i.e., including task-irrelevant ones; Brodeur & Pond, 2011).

In addition, our distractors’ potency may have been increased by their high salience, in terms of their visual appearance (e.g., color, size, visual complexity), cultural meaning, and positive emotional associations (e.g., because of our choice of sympathetic protagonist characters rather than villains). The infrequency of distractor presentation in our task may also play a key role. Notably, similar cartoon characters presented as high-frequency response-competition distractors have previously failed to produce inflated distraction in ADHD (Geurts et al., 2008). Future research should identify which of these distractor features are necessary to differentiate ADHD participants and controls.

We note that, although our sample size had sufficient statistical power to detect group differences in the level of distraction, larger scale studies are required to establish our measure as a valid and reliable distractibility test. In addition, future studies should clarify whether our measure predicts informant ratings of inattentive symptoms as closely as self-ratings. Finally, further investigations are still required to clarify the causes of distractibility in ADHD, including the role of both executive (e.g., inhibition) and nonexecutive (e.g., state regulation) factors (Johnson et al., 2009).

In summary, the present study makes three key contributions to the study of ADHD. Our findings introduce the irrelevant distractor paradigm as an objective and sensitive method for assessing distraction in ADHD. This method should prove useful in future research (e.g., assessing distraction in a variety of contexts, evaluating the efficacy of pharmacological and nonpharmacological treatments in reducing distraction in ADHD and so forth). In addition, we clarify that increased distraction in ADHD reflects disruption to only one of two mechanisms underlying the avoidance of distraction. Finally, we demonstrate a potentially powerful method for reducing the heightened levels of distraction associated with ADHD, by increasing perceptual load. This finding, with the somewhat counterintuitive implication that increasing, rather than decreasing, the perceptual difficulty of tasks for people with ADHD may in fact facilitate their performance, provides a promising direction for the development of novel interventions to overcome this disruptive component of the inattention of ADHD.

## Figures and Tables

**Table 1 tbl1:** Mean Age, WASI T-Scores, and Scores on the Barkley and Murphy’s (2006) Current Symptoms Rating Scale Symptom Count and Summary Score by Group (SE)

	Current symptoms							
		Symptom count		Summary Score		IQ		
	Age	Inattentive	Hyperactive-impulsive	Inattentive	Hyperactive-impulsive	Matrix reasoning	Vocabulary	Total
ADHD	34.65 (2.67)	8.11 (0.22)	6.47 (0.64)	21.12 (.70)	18.00 (1.42)	57.62 (2.62)	54.38 (2.37)	112.00 (4.12)
Control	32.88 (2.24)	1.18 (0.31)	1.06 (0.31)	6.31 (.86)	6.06 (.78)	60.24 (1.52)	52.41 (1.97)	112.64 (3.06)

**Table 2 tbl2:** Mean RTs and Percentage Error Rates (SE in Parentheses) as a Function of Group and Experimental Conditions

Distractor condition						
	Distractor		No distractor		Distractor cost (Distractor−No distractor)	
	ADHD	Control	ADHD	Control	ADHD	Control
Low load						
RT (ms)	681 (36)	558 (20)	580 (25)	515 (16)	101 (20)	43 (10)
% Error	11.94	9.75	8.00	6.38		
High load						
RT (ms)	905 (38)	794 (28)	845 (31)	778 (23)	60 (15)	16 (9)
%Error	29.82	17.31	22.29	17.56
